# Prediction of synergistic drug combinations using PCA-initialized deep learning

**DOI:** 10.1186/s13040-021-00278-3

**Published:** 2021-10-20

**Authors:** Jun Ma, Alison Motsinger-Reif

**Affiliations:** 1 Bioinformatics Research Center, North Carolina State University, Raleigh, NC USA; 2 Biostatistics and Computational Biology Branch, National Institute of Environmental Health Sciences, 111 TW Alexander Drive, Durham, NC 27709 USA

**Keywords:** Deep learning, Cancer treatment, Drug combination treatment, Feedforward neural network, Machine learning, XGBoost, Random Forests, Elastic net

## Abstract

**Background:**

Cancer is one of the main causes of death worldwide. Combination drug therapy has been a mainstay of cancer treatment for decades and has been shown to reduce host toxicity and prevent the development of acquired drug resistance. However, the immense number of possible drug combinations and large synergistic space makes it infeasible to screen all effective drug pairs experimentally. Therefore, it is crucial to develop computational approaches to predict drug synergy and guide experimental design for the discovery of rational combinations for therapy.

**Results:**

We present a new deep learning approach to predict synergistic drug combinations by integrating gene expression profiles from cell lines and chemical structure data. Specifically, we use principal component analysis (PCA) to reduce the dimensionality of the chemical descriptor data and gene expression data. We then propagate the low-dimensional data through a neural network to predict drug synergy values. We apply our method to O’Neil’s high-throughput drug combination screening data as well as a dataset from the AstraZeneca-Sanger Drug Combination Prediction DREAM Challenge. We compare the neural network approach with and without dimension reduction. Additionally, we demonstrate the effectiveness of our deep learning approach and compare its performance with three state-of-the-art machine learning methods: Random Forests, XGBoost, and elastic net, with and without PCA-based dimensionality reduction.

**Conclusions:**

Our developed approach outperforms other machine learning methods, and the use of dimension reduction dramatically decreases the computation time without sacrificing accuracy.

## Background

Cancer is the second leading cause of death globally and is a critical cause of economic burden throughout the world. According to estimates from the American Cancer Society, there were 17.0 million new cancer cases and 9.5 million cancer deaths worldwide in 2018 [1]. By 2040, new cancer cases and deaths are expected to grow to 27.5 million and 16.3 million [1]. The financial costs of cancer research are also increasing, primarily because the development of new pharmaceutical anticancer agents is labor- and cost-intensive due to the requirements for initial in vitro and in vivo experimentation and subsequent clinical trials to receive FDA approval [2–4]. Previous studies have suggested that it costs, on average, $2.8 billion for a pharmaceutical company to bring a newly designed drug to market, with the process taking up to 15 years [3, 4]. Therefore, it is important to find more efficient and economically feasible strategies to discover cancer treatments.

Compared with monotherapy treatments, combination drug therapy is more effective for treating complex diseases such as cancer, acquired immunodeficiency syndrome (AIDS), asthma, and hypertension [5–9]. Multi-agent therapies are preferable to monotherapy for many reasons. First, combination drug therapies can reduce host toxicity and adverse side effects since doses of drugs comprising multi-agent therapies are usually lower than doses of single drugs [10]. Additionally, drug combination therapies can overcome acquired drug resistance [11, 12]. As a result, combination therapies are rapidly becoming standard practice in cancer treatment. While there have been clear successes in this area, major challenges exist in finding synergistic combinations of drugs.

High-throughput screening using cancer cell lines is crucial to identify effective cancer combination therapies [13–16]. In these screens, drug pairs at different concentrations are applied to a cell line (typically a cancer line), and the combined effect of the drug pair is measured [14–16]. While in vitro or in vivo drug screening experiments are the standard method to discover synergistic drug combinations, the enormous number of possible drug combinations and economic and technical burdens make this process slow and expensive [8]. Exhaustively testing the huge number of possible combinations is impractical and motivates the need for computational approaches for predicting drug synergies.

Throughput experimental methods depend on computational modeling to measure and predict synergy. A wide variety of machine learning methods are currently emerging as powerful tools for efficiently exploring the large synergistic space with chemical, biological, and molecular data from cancer cell lines. Previous methods vary and include systems approaches and algorithms for the discovery of combinatorial therapies [17], mixed-integer linear programming [18], kinetic models [19], and machine learning approaches such as Naive Bayesian methods [20], Random Forests [21], and deep learning [22]. While these methods show promise, each has important limitations. For example, many systems biology and kinetic models are restricted to using information only from certain pathways based on hypotheses related to the mechanism of action, which are increasingly understood to be inaccurate [23]. Other machine learning approaches have limitations due to the exponential increase in the dimensionality of data [24].

Deep learning is a subfield of machine learning inspired by the neural networks that define the structure and function of the brain [25, 26]. Deep learning benefits from and performs well on large datasets, outperforming many traditional machine learning methods [25]. It has revolutionized the fields of computer vision [27], speech recognition [28], and natural language processing [29] and was recently used to predict synergistic drug combinations due to its ability to extract important features in large datasets [22]. However, the large number of features included in deep learning models significantly increases the associated computational complexity and time costs. Additionally, optimizing the large number of parameters in such models may cause overfitting problems although the magnitude and impact of overfitting are highly context-specific in regard to bias and variance tradeoff [30, 31].

**Fig. 1:**
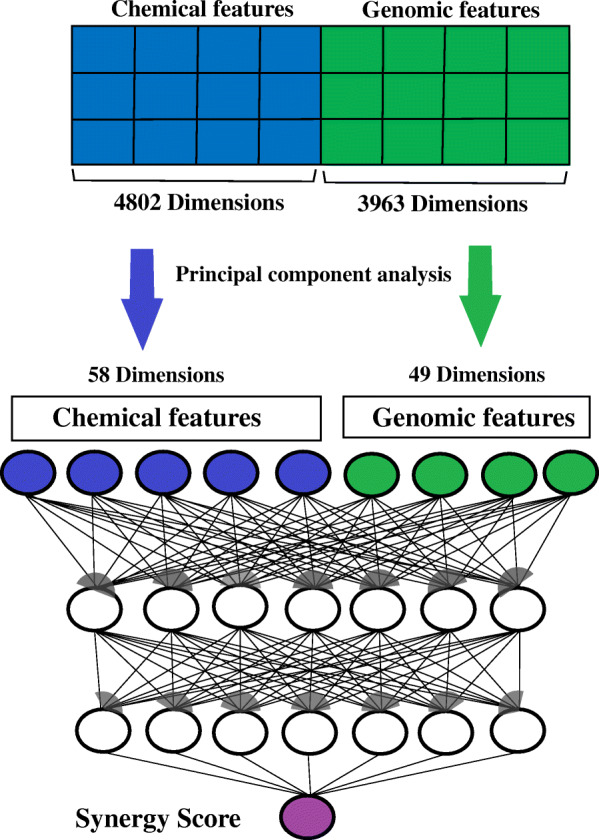
Flowchart of PCA-initialized deep learning for drug synergy prediction. PCA reduced the dimensionality of the chemical descriptor and gene expression data. The low-dimensional data were then propagated through the neural network to determine the drug synergy values

To address these concerns, we developed a new deep learning approach with dimensionality reduction to predict synergistic drug combinations by integrating gene expression profiles of cell lines and chemical structure data. Specifically, we used principal component analysis (PCA) to reduce the dimensionality of chemical descriptor data and gene expression data. PCA is one of the oldest and most extensively used approaches to reduce the dimensionality of large data sets by transforming a large set of variables into a smaller one that retains most of the information in the large set [32]. PCA works by finding the orthogonal vectors in a dataset that account for the greatest amount of variation, where each orthogonal vector is a linear combination of all the features in the original dataset. Subsequently, we propagated the low-dimensional data through the neural network to the linear output unit (drug synergy values). Figure 1 outlines the process of PCA-initialized deep learning for drug synergy prediction.

While PCA relies on linear combinations for dimensionality reduction, neural network-based reduction approaches can also be considered. We considered an alternate dimensionality reduction technique through an autoencoder to impose a bottleneck in the network, which forces a compressed knowledge representation of the original input [33]. Autoencoders can be considered a nonlinear generalization of PCA [34]; whereas PCA attempts to discover a lower-dimensional hyperplane that describes the original data, autoencoders are capable of learning nonlinear manifolds (in simple terms, a continuous, non-intersecting surface). We compared the use of PCA with an autoencoder strategy for dimensionality reduction within our framework.

As part of the current study, we evaluated our method on a range of simulated datasets and directly compared it with three other machine learning models. Additionally, we evaluated our method on two seminal drug-combination high-throughput screening datasets. Recently, O’Neil et al. from Merck & Co. performed a large-scale high-throughput drug-screening study with more than 20,000 drug synergy measurements [15]. This dataset comprises 23,062 samples, where each sample is one of 583 two-drug combinations tested in 39 cancer cell lines from different tissues of origin [15]. The second dataset was provided by the AstraZeneca-Sanger Drug Combination Prediction DREAM Challenge [14]. The DREAM Challenge data consists of 11,576 experimentally tested drug combinations of 118 targeted drugs on 85 cancer cell lines [14]. Molecular data for untreated cell lines, as well as drug chemical information, are also provided [14].

## Methods

### Experimental drug combination screening dataset

We used O’Neil’s high-throughput drug-combination screening data to train our models. Each of the dataset’s 23,062 samples consists of two compounds and a cell line [15]. We tested each of the dataset’s 583 distinct combinations against 39 human cancer cell lines derived from seven tissue types, namely lung, breast, skin, large intestine, pleura, prostate, and ovary. Unlike other high-throughput screening data, these data include unbiased cell lines from sites such as the breast, lung, and colon. The choice of drugs is also unbiased; drugs can be FDA-approved, chemical compounds used in clinical trials, chemotherapy drugs, or targeted therapy drugs. The pairwise combinations were constructed from 38 diverse anticancer drugs, 22 of which were tested exhaustively in combination with each other only (the ‘exhaustive’ set) while the remaining 16 were tested only in combination with drugs in the exhaustive set (the ‘supplemental’ set) . We assayed each sample according to a 4 × 4 dosing regimen in quadruple replicate to measure the rate of cell growth relative to a drug-free control after 48 h.

Additionally, we tested our model with a dataset from the AstraZeneca-Sanger Drug Combination Prediction DREAM Challenge [14]. The approximately 11,500 synergy scores provided by the DREAM consortium were experimentally assessed. We measured the synergistic effects through combinatorial in vitro drug screening of 85 cancer cell lines and 118 anonymous chemical compounds. The cancer cell lines come from breast (*n* = 34), lung (*n* = 22), urinary tract (*n* = 14), gastrointestinal tract (*n* = 12), male genital system (n = 2), and lymphoma (n = 1) tissue. In each drug screening experiment, we tested five nontrivial doses of each drug. We summarized the observed drug responses in a 6 × 6 matrix. We measured the efficacy of the drugs by the percentage of the cells remaining after drug treatment compared with the untreated cells.

### Synergy score

We employed the Combenefit tool [35] to quantify the synergy level between a drug pair and a cancer cell line using dose-response data. Combenefit enables model-based quantification of drug combinations by comparing additive and actual effects for given dose-response data. This tool calculates a synergy score, which is the difference between the Loewe model-based expected additive effect and the actual effect of the drug combination. If the actual effect of a drug combination is greater than the additive effect, the synergy score is higher than zero; otherwise, it is less than zero. A higher synergy score denotes greater synergy of the corresponding drug combination [36]. We calculated Loewe additivity values using the batch processing mode of Combenefit [37] and subsequently averaged the replicates, which resulted in a set of 22,737 (compound, compound, cell line, synergy value) quartets.

### Chemical descriptors and genomic features

For O’Neil’s high-throughput drug-combination screening data [15], drug chemical information and cell-line gene expression profiles were used as input features for the analysis. Approximately 46,000 drug pairs are described by chemical “fingerprints”. Computed by several online tools, these fingerprints describe the structure of the drug pairs, including charge, connectivity, and local features such as Morgan fingerprints [22]. We reduced the chemical feature space by filtering out features with zero variance. The final set of chemical features consists of 1309 extended connectivity fingerprints (ECFP), 6802 physicochemical features, and 2276 toxicophore features. Approximately 4000 genomic features represent the gene expression of the cell line on the drug-pair synergy scores that we experimentally measured. We measured gene expression levels on an Affymetrix Human Genome U219 array plate using Affymetrix HG-U219 arrays accessed from ArrayExpress (http://www.ebi.ac.uk/arrayexpress) with accession number E-MTAB-3610. The final gene expression dataset contains 3984 genomic features. In summary, the input dataset comprises 22,737 samples, and each sample has 8846 chemical or genomic features. The target is drug-pair synergy scores, so we combined these datasets to generate a set of 22,737 (compound, compound, cell line, synergy value) quartets.

For the DREAM Challenge data [14], the DREAM consortium provided drug target information and physicochemical properties of 118 drugs as well as Genomics of Drug Sensitivity in Cancer (GDSC) gene expression data from Affymetrix Human Genome U219 array plates (E-MTAB-3610).

### Dimensionality reduction

The high dimensionality of the feature space is the main difficulty encountered when constructing a model to predict synergism. To address this, the number of genomic features such as mutations and expressions of more than 10,000 genes need to be substantially reduced. To accomplish this, we reduced the feature dimensions with PCA, a technique to decrease the dimensionality of a dataset comprising multiple variables with light or heavy correlation while retaining the variation present to the maximum extent. In this study, we implemented PCA in the scikit-learn Python package [38] and reduced the dimension of the input datasets while retaining 99% of the variance. The features for each sample are represented as a 107-dimension vector. Additionally, we compared the performance of PCA with autoencoder, another popular dimensionality reduction technique, also implemented in the scikit-learn Python package [38]. While PCA is a linear reduction approach, autoencoder enforces constraints on the neural network with non-linear activation functions. If we were to construct a linear network (i.e., without the use of nonlinear activation functions at each layer), we would observe a similar dimensionality reduction approach as in PCA.

### Feedforward neural network

The feedforward neural network models were trained on a preprocessed dataset using Keras, an open-source neural network library written in Python [39]. Feedforward neural networks represent a powerful deep learning method and are gaining popularity due to their superior accuracy when trained with large data. The difficult process of identifying new drugs includes many challenges for which deep learning is perfectly suited, for example, large amounts of available data and the technique’s ability to address complex tasks [40], including non-linear interactions in the ‘omics' space [41]. The feedforward neural network maps input vectors representing samples to a single output value—the synergy score. The samples are described by concatenated vectors that include the features of two drugs and one cell line. The neurons in the input layer receive the gene expression values of the cell line and chemical descriptors of both drugs as inputs. The information is then propagated through the layers of the network until reaching the output unit, which produces the predicted synergy score. To determine the best hyperparameters for each model, we tuned the models using grid search. Table 1 shows the hyperparameter settings considered for the feedforward neural network. For data normalization, we employed three types of input normalization: (1) standardizing all inputs to zero mean and unit variance, (2) standardizing and applying the hyperbolic tangent, and (3) standardizing as in (1), applying the hyperbolic tangent as in (2), and standardizing again as in (1). The hidden layers apply rectified linear activation, and the output layer uses linear activation. The mean squared error (MSE) is the objective function that is minimized.

**Table 1:** Hyperparameter settings considered for the feedforward neural network

Hyperparameter	Values considered
Preprocessing	norm; norm+tanh; norm+tanh+norm
Hidden units	[107, 107]; [54, 54]; [27]; [107,54]; [54,27]; [54, 54, 54]; [27]; [54, 27, 13]; [107, 54, 27]
Learning rates	10^−2^; 10^−3^; 10^−4^; 10^− 5^
Dropout	no dropout; input: 0.3, hidden: 0.5

### Comparison of the feedforward neural network with other machine learning methods

We compared the performance of the feedforward neural network with the elastic net [42], Random Forests [43], and eXtreme Gradient Boosting package (XGBoost) [44] methods used in previous studies for drug synergy prediction. These methods represent two of the most commonly used machine learning algorithms—regularization algorithms and ensemble algorithms—both of which have good performance when solving regression problems.

Elastic net linear regression uses the penalties from the lasso and ridge techniques to regularize regression models [42]. The elastic net technique addresses the shortcomings of other techniques to improve on the regularization of statistical models. To eliminate the limitations of lasso, the elastic net method includes a quadratic expression (||β||^2^) in the penalty, which, when used in isolation, becomes ridge regression [42]. The quadratic expression in the penalty elevates the loss function toward convexity. The elastic net method draws on the best aspects of lasso and ridge regression and has good performance for highly correlated variables.

The Random Forests method used for modeling predictions and behavior analysis is built on decision trees [43]. Multiple decision trees represent a distinct instance of the data input into the random forest. The technique individually considers instances, selecting the instance with the majority of votes as the prediction [43]. The technique is not computationally expensive, does not require a GPU to finish training, and generally produces a robust model. Compared with Random Forests, neural networks require much more data and are computationally more expensive but usually perform better.

XGBoost is a decision-tree-based ensemble machine learning algorithm that employs a gradient boosting framework [44]. XGBoost supports objective functions that include regression, classification, and ranking. In prediction problems, neural networks tend to outperform other algorithms and frameworks. However, for small to medium structured/tabular data, decision-tree-based algorithms such as XGBoost are considered the best option [44].

In this study, we consider different numbers of estimators (trees) and tune the number of features in each split for Random Forests. We also consider different values for α and the L1 ratio during elastic net hyperparameter selection [42] and different learning rates for XGBoost [44]. Tables 2, 3 and 4 summarize the hyperparameters considered for these methods.

**Table 2:** Hyperparameters considered for elastic net

Hyperparameter	Values considered
Preprocessing	norm; norm+tanh; norm+tanh+norm
α	0.1; 1; 10; 100
L1 ratio	0.25; 0.5; 0.75

**Table 3:** Hyperparameters considered for Random Forests

Hyperparameter	Values considered
Preprocessing	norm; norm+tanh; norm+tanh+norm
Number of estimators (decision trees)	128; 512; 1024; 2048
Features considered	Log2(# of features); sqrt(# of features); auto

**Table 4:** Hyperparameters considered for XGBoost

Hyperparameter	Values considered
Preprocessing	norm; norm+tanh; norm+tanh+norm
Number of estimators (decision trees)	128; 512; 1024; 2048
Learning rates	1; 0.1; 0.01

We implemented these three machine learning methods in scikit-learn, a machine learning library for Python [38]. We used grid search to select the best hyperparameters. We compared the predictive performance of the feedforward neural network, elastic net [42], Random Forests [43], and XGBoost [44] in terms of MSE, *P*-values, and Pearson’s r. In addition to implementing the machine learning methods without dimensionality reduction, we compared each method using data reduced using PCA to evaluate whether performance differences are attributable to the dimensionality reduction step or to the performance of the respective machine learning methods.

## Results

### Synergy score

**Fig. 2:**
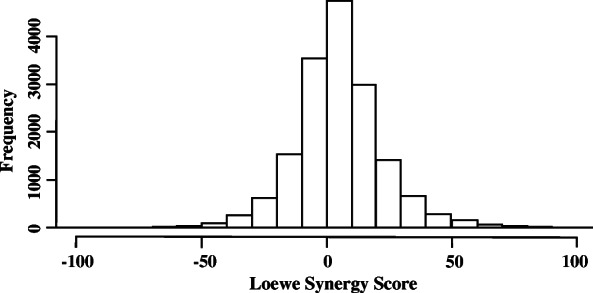
Distribution of synergy scores across all drug combinations. The synergy scores are Loewe additivity values calculated using Combenefit. If the effect of a drug combination is greater than the additive effect, the synergy score is > 0; if the effect of a drug combination is less than the additive effect, the synergy score is  < 0. A higher synergy score denotes greater synergy of the corresponding drug combination

We used O’Neil’s high-throughput drug combination screening data to train our models [15]. We employed the Combenefit tool to quantify the synergy level between two drugs and a cancer cell line using dose-response data. If the actual effect of a drug combination is greater than the additive effect, the synergy score is greater than zero; otherwise, it is less than zero. A higher synergy score denotes greater synergy of the corresponding drug combination. Figure 2 is a histogram of the distribution of synergy scores across all drug combinations. The average synergy score is 4.52, and the standard deviation is 20.65. The synergy scores obtained using Combenefit are target variables in the deep learning model used in the study.

### Architecture of the feedforward neural network

The architecture of the deep learning model was determined by the hyperparameter selection procedure. This procedure identified that tanh normalization, comprising standardization and then a hyperbolic tangent followed by a second standardization, performed the best. The model has conic layers with good performance, possibly due to their regularizing effect. Fewer parameters are available in the higher layers, which forces the model to generalize by constructing only the most important representations of chemical properties of the input drug combination. Additionally, the model had better performance with a large number of units in the first layer (107). A smaller learning rate (10^− 5^) and dropout regularization were essential for learning networks. In summary, the model has a conic architecture comprising two hidden layers, with 107 neurons in the first layer and 54 in the second. It uses tanh input normalization and has a learning rate of 10^− 5^, an input dropout rate of 0.2, and a hidden layer dropout rate of 0.5.

### Predictive performance

**Fig. 3:**
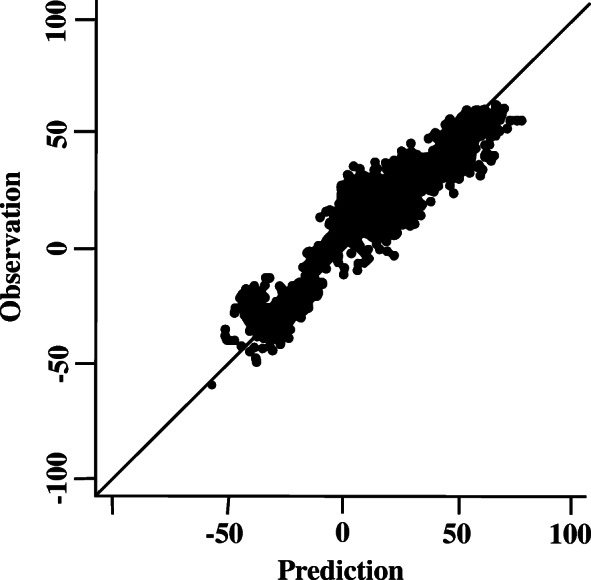
Scatterplot of observed synergy scores vs. predicted synergy scores. For most of the tested cell lines, the drug pair predicted to be the most synergistic is among the top-five synergistic drug combinations observed in the experimental data

**Fig. 4:**
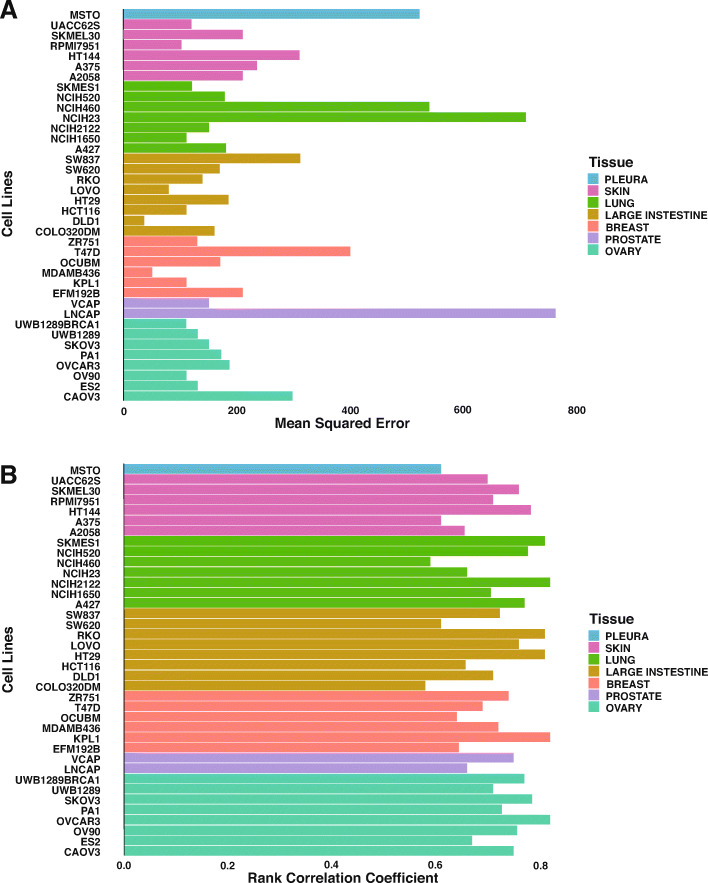
**A**) Rank correlation coefficients between observed and predicted synergy scores per cell line. The rank correlation coefficients vary from 0.56 to 0.81. Three cell lines have a rank correlation coefficient < 0.6, and over 55% of the cell lines have a rank correlation coefficient > 0.7. **B**) MSE of predicted synergy scores per cell line. The MSE varies from 61 to 526, and three cell lines have MSEs > 400

**Fig. 5:**
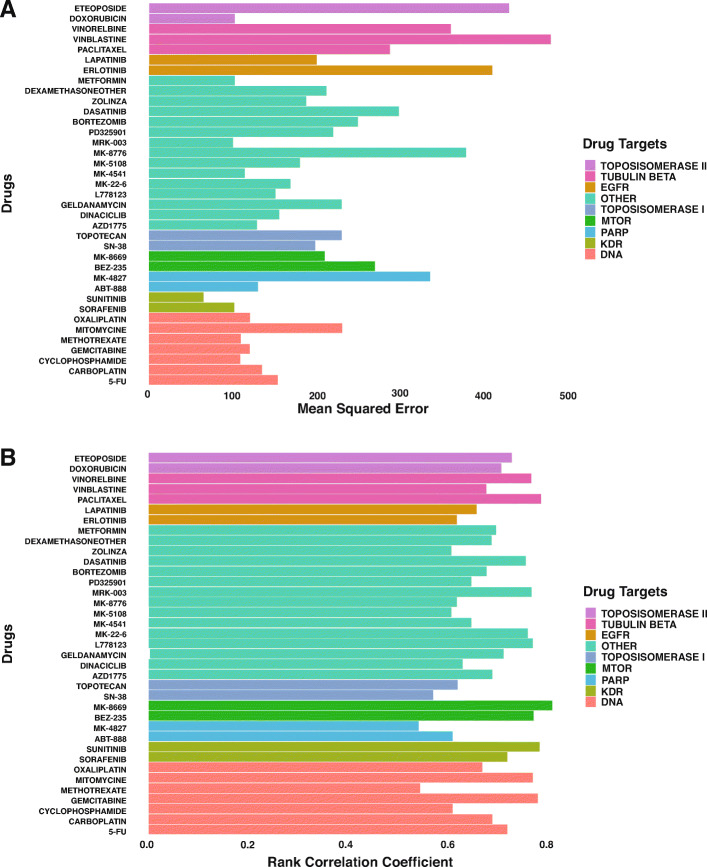
**A**) Rank correlation coefficients between observed and predicted synergy scores per drug. The rank correlation coefficients range from 0.55 to 0.86. Two drugs have rank correlation coefficients < 0.6, and 67% of the drugs have a rank correlation coefficient > 0.7. **B**) MSE of predicted synergy scores per drug. The MSEs for individual drugs vary from 64 to 846. Three drugs have MSEs > 400, and approximately 67% of the drugs have MSEs < 200

Figure 3 is a scatter plot that visualizes the correlation of the observed vs. the predicted synergy scores. The correlation coefficient is 0.761. We also analyzed the performance of the deep learning approach across cell lines and drugs by determining the respective rank correlation coefficients. The prediction accuracy varies significantly among cell lines and drugs, which may be due to the distinct mechanisms of different drug pairs’ synergistic/antagonistic effects on specific cell lines. As shown in Fig. 4B, for individual cell lines, the rank correlation coefficients vary from 0.56 to 0.81. The rank correlation coefficient is < 0.6 for three cell lines; for more than 55% of the cell lines, the correlation coefficient is > 0.7. As shown in Fig. 4A, the MSE varies from 61 to 526. Similarly, three cell lines have MSEs > 400, and for 50% of the cell lines, the correlation coefficient is < 0.7. As shown in Fig. 5B, the rank correlation coefficients for individual drugs range from 0.55 to 0.86. Two drugs have rank correlation coefficients < 0.6, and approximately 67% of the drugs have rank correlation coefficients > 0.7. As shown in Fig. 5A, the MSEs for individual drugs vary from 64 to 846. Three drugs have MSEs > 400, and approximately 67% of the drugs have MSEs < 200.

Moreover, we ranked the observed synergy scores and compared them with the drug pairs with the highest predicted scores. For about 96% of the tested cell lines, the drug pair predicted to be the most synergistic is among the top-five synergistic drug combinations according to the experimental data. For around 72.2% of the cell lines, the model can correctly predict the most synergistic pairs. Table 5 summarizes this result.

**Table 5:** Predicted ranks of the best drug pairs

Rank	Percentage
1	72.2%
2	8.0%
3	4.1%
4	6.0%
5	5.7%
> 5	4.0%

### Comparison of the PCA and autoencoder methods

PCA and autoencoder are commonly used dimensionality reduction techniques. The autoencoder method represents a branch of neural networks and attempts to compress information on the input variables into a reduced dimensional space and then recreate the input dataset. We compared the performance of the PCA and autoencoder techniques, as outlined in Table 6. The results suggest that for drug synergy prediction, PCA has better performance than the autoencoder method.

**Table 6:** Comparison of the PCA and autoencoder methods

Method	MSE	RMSE	Pearson’s *r*
Deep learning (PCA)	240.6 ± 38.44	15.14 ± 1.82	0.77 ± 0.02
Deep learning	254.6 ± 41.32	15.95 ± 1.57	0.73 ± 0.04
Deep learning (autoencoder)	298.06 ± 48.61	17.26 ± 1.53	0.68 ± 0.02

### Comparison of the methods with experimental data

We compared the methods’ ability to predict synergy values of novel drug combinations. The models were optimized for MSE, the primary metric, during training. Table 7 reports the performance of the methods based on the MSE, root mean square error (RMSE), and Pearson correlation coefficient for validation drug combinations (that were not used in training the model).

**Table 7:** Comparison of methods using O’Neil’s high-throughput drug combination screening data

Method	MSE	RMSE	Pearson’s *r*
PCA + Deep learning	241.20 ± 43.50	15.46 ± 1.44	0.76 ± 0.03
Deep learning	255.50 ± 49.54	15.91 ± 1.56	0.74 ± 0.04
PCA + Random Forests	319.80 ± 50.62	17.88 ± 1.31	0.55 ± 0.03
PCA + XGBoost	341.86 ± 49.71	18.49 ± 1.42	0.46 ± 0.02
PCA + Elastic net	362.48 ± 52.18	19.04 ± 1.38	0.41 ± 0.02

As shown in Table 7, deep learning with PCA achieved an MSE of 241.20 while Random Forests [43], XGBoost [44], and elastic net [42] achieved relatively inferior performance with MSEs of 319.80, 341.86, and 362.48, respectively. Additionally, as shown in Table 8, deep learning with PCA achieved similar performance with significantly reduced execution time compared with deep learning without PCA, suggesting that it is necessary to conduct dimensionality reduction before running deep learning.

**Table 8:** Execution time of deep learning (PCA) vs. deep learning

Method	Runtime
Deep learning (PCA)	43 min 22 s
Deep learning	46 h 21 min

Additionally, we evaluated the predictive performance of these methods on a separate dataset from the AstraZeneca-Sanger Drug Combination Prediction DREAM Challenge [14]. Table 9 shows the results, which exhibit similar trends.

**Table 9:** Comparison of methods using the DREAM Challenge dataset

Method	MSE	RMSE	Pearson’s *r*
PCA + Deep learning	279.30 ± 47.62	16.71 ± 1.30	0.56 ± 0.02
PCA + XGBoost	376.14 ± 51.27	19.39 ± 1.46	0.43 ± 0.04
PCA + Random Forests	395.18 ± 54.39	19.87 ± 1.28	0.39 ± 0.02
PCA + Elastic net	501.29 ± 58.37	22.39 ± 1.58	0.31 ± 0.03

## Discussion

In this study, we present a new deep learning approach with dimensionality reduction to predict synergistic drug combinations by integrating gene expression profiles of cell lines and chemical structure data. Specifically, we use PCA to reduce the dimensionality of chemical descriptor data and gene expression data and then propagate the low-dimensional data through the neural network to the linear output unit (drug synergy values). Our results suggest that deep learning outperforms three other machine learning methods in terms of predictive accuracy and stability.

The novelty of our proposed work lies in the use of PCA to reduce a dataset’s dimensionality before running deep learning. Among the disadvantages of deep learning is that it is computationally expensive and requires large amounts of processing power. Network training converges faster if its inputs are whitened (i.e., linearly transformed to have zero means and unit variances) and decorrelated. PCA yields decorrelated vectors, and subtracting the mean and rescaling by the standard deviation achieves the final whitened results, giving the model a boost in terms of training time. Further, leaving uninformative features in an analysis can lead to biased estimates and can decrease its power. In addition, reducing the dimensionality of the dataset may prevent overfitting issues. According to our results, the predictive performance of deep learning with PCA is slightly better than deep learning without PCA. However, in terms of execution time, deep learning with PCA runs 60 times faster than without PCA. Additionally, results from our comparisons with an autoencoder dimensionality reduction approach support the superior performance of PCA.

Various directions can improve and extend our PCA-initialed deep learning models. Most importantly, we plan to apply it to drug combination screens from primary patient-derived cancer cells. Such a model would better represent clinical cases and would increase the model’s potential for application in precision medicine. Moreover, we plan to integrate multi-omics data to improve the model’s predictive performance.

It is important to note that while our approach improves predictive performance in drug screening data, this does not equate to guarantees in regard to the translatability of findings [14, 45]. Screening exercises are limited by the drug and chemical class representations tested and do not represent or reflect in vivo challenges, which may limit translation of the findings to practice [45]. Further, pharmacokinetics and pharmacodynamics are not fully modeled by cell lines, which could limit efficacy or result in drug-drug toxicities [46].

## Data Availability

The dataset(s) supporting the conclusions of this article are available at the Synapse database [https://www.synapse.org/DrugCombinationChallenge] and the AstraZeneca Open Innovation Portal [https://openinnovation.astrazeneca.com/data-library.html].
